# Comparative Effects of Silkworm Excrement Concentrate Extract Versus Sodium Copper Chlorophyllin on Growth, Metabolic Health and Immune Response in Common Carp (*Cyprinus carpio*)

**DOI:** 10.3390/ani16030455

**Published:** 2026-02-01

**Authors:** Jiafa Yang, Shanren Lan, Xu Jia, Yaowei He, Zhijun Li, Aiguo Zhou, Huijuan Tang

**Affiliations:** College of Marine Sciences, South China Agricultural University, Guangzhou 510642, China; yangjiafa@stu.scau.edu.cn (J.Y.); 19978331043@163.com (S.L.); jxxx4018@163.com (X.J.); 17817833484@163.com (Y.H.); pz9333501@163.com (Z.L.)

**Keywords:** common carp, silkworm excrement, sodium copper chlorophyllin, growth performance, antioxidant capacity, lipid metabolism

## Abstract

This study investigated the efficacy of two value-added products derived from silkworm excrement—a concentrated extract (SCE, 20:1) and sodium copper chlorophyllin (SCC)—as functional feed additives for common carp. Diets supplemented with 0.5% SCE, 1.0% SCE, or 0.1% SCC were compared to a basal control. The results revealed a distinct dose-dependent effect for SCE: 0.5% SCE was safe, while 1.0% SCE impaired growth, feed efficiency, and digestive enzyme activity. Both SCE and SCC significantly enhanced lipid metabolism, reducing hepatic lipid deposition and improving serum lipid profiles, albeit through distinct molecular pathways—SCC primarily stimulated catabolism, whereas SCE comprehensively regulated both synthesis and breakdown. Furthermore, SCE demonstrated superior, multi-targeted immunomodulatory capacity by favorably regulating inflammatory cytokine expression, an effect not observed with SCC. Although both additives boosted systemic antioxidant capacity, their specific patterns of enzyme activity and gene expression differed. In conclusion, SCE offers broad-spectrum, synergistic benefits for health modulation, while SCC provides specific, stable bioactivity, highlighting the importance of selecting the appropriate additive form based on desired functional outcomes in aquaculture.

## Simple Summary

This study evaluated two silkworm-excrement-derived additives—SCE (concentrated extract) and SCC (sodium copper chlorophyllin)—in common carp feed. Results indicated that 0.5% SCE was safe, whereas 1.0% SCE impaired growth. Both additives enhanced lipid metabolism and antioxidant capacity but through different pathways. SCE provided broad-spectrum benefits, including multi-gene-mediated immunomodulation, while SCC exerted more targeted effects, primarily in lipid catabolism and selective antioxidant activity, with limited impact on immunity and growth. These findings demonstrate that multi-component SCE acts synergistically for integrated health modulation, while SCC serves as a specific, stable alternative. The study supports the valorization of agricultural by-products for sustainable aquaculture.

## 1. Introduction

The global shift towards sustainable agriculture necessitates a transition from environmentally detrimental practices to eco-friendly alternatives [1]. This transition creates an urgent demand for novel feed additives that synergistically integrate nutritional benefits, health promotion, and environmental sustainability. The mulberry dike and fish pond (MDFP) system, an ancient circular agriculture model recognized by the FAO as a global heritage [2], offers valuable insights through its closed-loop design. A key by-product of this system is silkworm excrement (SE), with an annual output exceeding several million tons [3]. Underutilization of SE not only represents a significant waste of resources but also poses environmental risks. However, SE is rich in protein, essential minerals, and bioactive compounds such as flavonoids and chlorophyll [3,4]. This unique composition elevates it from a mere fertilizer to a promising functional feed additive for aquaculture.

As a traditional Chinese medicine, SE has a long history, which was recorded in the Compendium of Materia Medica [5]. Modern pharmacological studies have further substantiated its bioactivities, demonstrating efficacy in alleviating alcoholic liver injury and reducing serum lipids in rats [6], exerting anti-diabetic effects through the AMPK/PI3K/Akt pathway [7], and enhancing antioxidative capacity in murine models of anemia [8]. The bioactivities of SE also suggest potential applications in aquaculture; preliminary studies in grass carp (*Ctenopharyngodon idellus*) indicate that a 10% dietary inclusion of SE can improve muscle texture and quality [9]. Furthermore, a 10% inclusion of fermented SE has been shown to enhance non-specific immunity. However, this benefit is dose-dependent, as inclusion levels exceeding 15% impair growth, digestive capacity, and intestinal health [10]. SE has advantages as a feed additive, but its direct use requires high inclusion levels. This dilutes nutrients, occupies formula space, and offers low active ingredient utilization. Therefore, using an SE concentrated extract (SCE, 20:1) at a low inclusion rate is a better strategy.

It is worth noting that SE is often used to prepare sodium copper chlorophyllin (SCC) [11]. SCC is synthesized via chlorophyll saponification, wherein magnesium is replaced by copper and hydrophobic side chains are removed, conferring superior stability [12]. In contrast to natural chlorophyll, which denatures into pheophytin above 60 °C, SCC remains stable throughout the production of aquatic pellet feed, which typically involves processing at approximately 80 °C [13,14,15]. Furthermore, pharmacological studies have demonstrated that SCC retains antioxidant properties comparable to those of chlorophyll and exhibits hepatoprotective effects in diabetic mouse models [16]. Therefore, comparing multi-component SCE with its purified derivative, SCC, will help deepen our understanding of both substances. To this end, this comparative approach can provide key insights for developing targeted, effective, and sustainable feed additives from SE.

China is both the largest producer and consumer of common carp [17], with annual production expanding from 3.42 million tons in 2012 to 4.24 million tons in 2020 [18], establishing common carp’s commercially significant status in the aquaculture sector. However, the widespread adoption of intensive aquaculture has boosted fish production while compromising fish health [19]. Concurrently, the elevated incidence of diseases under such farming systems has led to excessive antibiotic dependency among fish farmers [20]. For this problem, developing new functional feed additives [21] is one of the measures to address these issues.

Based on the aforementioned background, this study aims to systematically compare the effects of two value-added products derived from silkworm excrement—multi-component SCE and the purified compound SCC—as functional feed additives. Using an economically important fish species as the model, the research will evaluate the impacts of different inclusion levels of SCE (e.g., 0.5% and 1%) and an appropriate SCC group on key performance indicators such as growth performance, antioxidant capacity, immune response, and lipid metabolism. Furthermore, the expression levels of relevant genes will be examined to elucidate the underlying regulatory mechanisms. Ultimately, this work seeks to clarify whether the synergistic activity of multiple bioactive components in SCE or the defined stability and efficacy of SCC offer greater advantages in practical feed formulations. The findings are expected to provide critical evidence for developing efficient and environmentally friendly feed additives, thereby promoting the high-value utilization of SE and supporting the sustainable development of aquaculture.

## 2. Materials and Methods

### 2.1. Feed Design and Preparation

SCE was purchased from Shaanxi Health Biotech Co., Ltd. (Xian, China), and SCC (95%) was supplied by Zhengzhou Xuanran Biotech Co., Ltd. (Zhengzhou, China). Other feed ingredients were provided by Xinhui Xinjian Feed Co., Ltd. (Jiangmen, China). Four experimental diets (Table 1) with varying dry-weight ratios of SCE to SCC were assigned to the following groups: no addition (CT) and addition of 0.1% SCC (SCC01), 0.5% SCE (SCE05) and 1.0% SCE (SCE10). Sodium carboxymethyl was used to balance the different contents of SCC and SCE, and all ingredients were crushed, ground and mixed with water. Then, they were extruded into strips using a twin-screw extruder at an appropriate speed, subsequently cut into particles and sieved through a 2.5 mm sieves. Subsequently, the diets were stored in a sealed container at −20 °C to minimize fatty acid oxidation until needed.

### 2.2. Experimental Trial

All juvenile common carp used for breeding experiments complied with the “Guiding Principles in the Care and Use of Animals (China)”, and the experiment was reviewed and approved by the Animal Welfare Division of South China Agricultural University in Guangzhou, China (2024G038).

All juvenile common carp used in this experiment were purchased from Guangzhou Guangyuan aquatic Development Co., Ltd. (Guangzhou, China). Prior to the experiment, the juvenile common carp had already acclimated to the recirculating aquaculture system at the College of Marine Sciences, South China Agricultural University, and were domesticated and fed with the CT group’s diet for one week. In accordance with ethical guidelines for experimental fish, 360 healthy juvenile fish (average body weight: 1.99 ± 0.05 g) were selected and randomly allocated into four experimental groups, each maintained in triplicate tanks (*n* = 3 replicates per group). These groups were then placed into 12 glass tanks (each with a 150 L capacity with oxygenation pumps for continuous aeration), with each tank containing 30 juvenile common carp. All juvenile common carp were fed to satiation twice a day (at 9:30 a.m. and 4:30 p.m. daily), and the daily intake was recorded. The juvenile common carp feeding experiment lasted for 12 weeks with water quality parameters of water temperature 26.0 ± 2.5 °C, dissolved oxygen above 7.00 mg/L and pH 6.70–7.50. Nitrite nitrogen and ammonia nitrogen in the recirculating aquaculture system were measured weekly to ensure that their concentration remained below 0.1 mg/L.

### 2.3. Sampling

Following the 12-week feeding trial, the fish were sampled after 24 h of fasting. The total number, length and weight of the fish in each tank were recorded before sampling. For proximate composition analysis, three fish per tank were randomly selected and stored at −20 °C for whole-body analysis, while from another three fish per tank, the livers were collected and stored at −20 °C prior to hepatic composition analysis. Following anesthesia (pentobarbitone 1:10,000) [22], blood samples were collected from the caudal vein of six fish per tank using a 1 mL syringe and transferred into 1.5 mL Eppendorf tubes. After blood collection, the same fish were dissected. The visceral mass (including liver and intestine) was excised and weighed to determine the viscerasomatic index. Subsequently, the liver and intestine were carefully separated from the visceral mass. The liver was weighed individually for the calculation of the hepatosomatic index. Finally, the liver, intestine, and the remaining visceral tissues were collected and stored at −80 °C for subsequent enzymatic activity assays. For serum preparation, the blood samples were centrifuged at 4000 rpm for 10 min at 4 °C. The obtained serum was aliquoted and stored at −80 °C until analysis for serum enzyme activities. For histological examination, three additional fish per tank were anesthetized. Liver and midgut samples were collected and immediately fixed in 4% paraformaldehyde. For each experimental parameter, measurements were conducted with six biological replicates per treatment group (*n* = 6; two fish pooled per biological replicate), and each biological sample was analyzed in triplicate. The remaining sample aliquots were preserved for contingency purposes. The entire sampling procedure described above was performed with reference to the method of Liao et al. [22].

### 2.4. Growth Performance

According to the data obtained during breeding and sampling, the growth parameters of experimental fish were calculated by the following formula, as described in Liao et al. [22]:

(1) Food intake (FI, %/day) = Feed consumed (dry weight) × 100 × 2/[84 × (Initial fish weight + Final fish weight + Dead fish body weight)];
(2) Weight gain (WG, %) = [(Final fish weight − Initial fish weight)/Initial fish weight] × 100;
(3) Specific growth rate (SGR, %/day) = 100 × [Ln (Final fish weight) − Ln (Initial fish weight)]/84 days;
(4) Feed conversion ratio (FCR) = Feed intake (dry weight)/(Final fish weight − Initial fish weight);
(5) Survival rate (SR, %) = (Final fish number/Initial fish number) × 100;
(6) Condition factor (CF, %) = [Fish body weight/(Length of fish)^3^] × 100;
(7) Hepatosomatic index (HSI, %) = (Weight of liver/Fish body weight) × 100;
(8) Viscerasomatic index (VSI, %) = (Weight of viscera/Fish body weight) × 100;
(9) Protein efficiency rate (PER, %) = 100 × (final fish weight − initial fish weight)/(total feeding dry weight × crude protein content in feed).

### 2.5. Proximate Composition Assay

The proximate, composition including moisture, crude protein, crude lipid and ash in experimental diets, whole fish and the liver, were determined based on wet weight. Before the determination of proximate compositions, the samples were oven-dried at 105 °C to a constant weight, grounded and mixed for homogeneity. The determination method is based on the Chinese national standard analysis method (P.R.C. GB/T), including moisture (P.R.C. GB/T 6435-2014), crude protein (P.R.C. GB/T 6432-2018), crude lipid (P.R.C. GB/T 6433-2025) and crude ash (P.R.C. GB/T 6438-2007) [23,24,25,26]. The instruments used in the experiment included a semiautomatic instrument (Buchi, Flawil, Switzerland), Soxhlet extractor (VELP Scientific, Milano, Italy) and Thermolyne muffle furnace (Thermo Fisher Scientific, Pittsburg, PA, USA), etc. For each experimental group, assays were conducted with six biological replicates (*n* = 6; two fish per replicate tank) and three technical replicates per biological sample.

### 2.6. Biochemical Indices and Enzymatic Activities Analysis

Serum, hepatic, and intestinal biochemical indices and enzymatic activities were determined using commercial diagnostic kits (Nanjing Jiancheng Bioengineering Institute, Nanjing, China). Specifically, serum samples were assayed for triglyceride (TG, A110-1-1), total cholesterol (TC, A111-1-1), high-density lipoprotein cholesterol (HDL-C, A112-1-1), low-density lipoprotein cholesterol (LDL-C, A113-1-1), total protein (TP, A045-2-2), albumin (ALB, A028-2-1), lysozyme (LZM, A050-1-1), aspartate aminotransferase (AST, C010-2-1), and alanine aminotransferase (ALT, C009-2-1). Hepatic samples were analyzed for total protein (TP, A045-2-2), total antioxidant capacity (T-AOC, A015-2-1), malondialdehyde (MDA, A003-1-2), superoxide dismutase (SOD, A001-3-2), catalase (CAT, A007-1-1), and lipase (LPS, A054-2-1). Intestinal samples were assessed for total protein (TP, A045-2-2), trypsin (TPS, A080-2-2), lipase (LPS, A054-2-1), and amylase (AMS, C016-1-1). To determine the enzyme activity of the liver and intestine, the samples were mixed with ice-cold and sterile physiological saline (1:9 *w*/*v*) in the tissue grinding tube, steel balls were added, and the samples were placed in a High-Throughput Tissue Homogenizer (Xinyi-48, Ningbo Xinyi Ultrasonic Equipment Co., Ltd., Ningbo, China) for mechanical homogenization; subsequently, the homogenates were then centrifuged at 4 °C (Desktop High-Speed Refrigerated Centrifuge, TG16-Ws, Hunan Xiangyi Laboratory Instrument Development Co., Ltd., Hunan, China) at speeds specified by the respective kit instructions. The resulting supernatant was collected as a 10% tissue homogenate. For serum samples, the serum was appropriately diluted on ice with ice-cold physiological saline based on preliminary experiments, thoroughly vortex-mixed, and then used for the subsequent assay. The 10% tissue homogenate and serum were treated by the determination method and reagent of the diagnostic kit and transferred to 96-well microplates (Synergy Multimode Reader, Winooski, VT, USA), the absorbance was read with MicroplateReader as required, and the enzyme concentration was calculated according to the absorbance. Non-HDL-C was calculated by subtracting HDL-C from TC, while the HDL/LDL ratio was derived from dividing HDL-C by LDL-C. For each experimental group, assays were conducted with six biological replicates (*n* = 6; two fish per replicate tank) and three technical replicates per biological sample.

### 2.7. Histology Analysis

Liver samples fixed with 4% paraformaldehyde were dried, paraffin-embedded, sectioned, and hematoxylin and eosin (H&E)-stained by Wuhan Servicebio Technology Co., Ltd. (Wuhan, China) using standard tissue sectioning techniques. Following preliminary screening with a digital scanning microscopy imaging system (M8, PreciPoint Co., Garching, German), detailed observation was specifically focused on liver histology using a Nikon Eclipse Ci-Light microscope (OLYMPUS, CX23, Nikon Co., Tokyo, Japan) equipped with an advanced imaging system. Observations and image acquisition were conducted at a final magnification of 400×, enabling the evaluation and documentation of specific tissue features. Based on these images, the density of lipid vacuoles in the liver was quantified using the ImageJ 1.51j8 software, according to the method described by Su et al. [27].

### 2.8. Quantitative Real-Time PCR Analysis

Real time PCR was used to detect the expression of genes in the liver of experimental fish. The related genes include lipid metabolism, immunity, antioxidation and glucose metabolism. The experimental process included the extraction of RNA from the sample, the synthesis of cDNA, and the amplification of target genes using the designed primers and the related enzyme. The kits and instruments used in the experiment included a total RNA extraction kit (model 9767, Takara, Tokyo, Japan), PrimeScript TMRT reagent kit (product code RR0047A, Takara, Tokyo, Japan), SYBR Green Master Mix (batch QPS-201T, Toyobo, Osaka, Japan), SYBR Green Master Mix (batch QPS-201T, Toyobo, Osaka, Japan) and a CFX Connect RealTime System (manufactured by Bio-Rad Laboratories, Hercules, CA, USA). The primers (Table 2) used in the experiment refer to the designed primers in the published papers involving common carp. The relative expression levels of all target genes were calculated by 2^−∆∆CT^ method. For each experimental group, assays were conducted with six biological replicates (*n* = 6; two fish per replicate tank) and three technical replicates per biological sample. RNA integrity was first assessed by 1% agarose gel electrophoresis. The purity of the RNA samples was then evaluated by measuring their absorbance at 260 nm and 280 nm. An OD_260_/OD_280_ ratio ranging from 1.8 to 2.0 was considered indicative of high-quality RNA, and only samples meeting this criterion were used for subsequent analyses. *β-Actin* was used as the internal reference gene for normalization. The qPCR methodology was performed with reference to Yang et al. [28].

### 2.9. Statistical Analysis

Statistical analysis was performed using SPSS (version 26.0). After confirming the assumptions of normality and homogeneity of variance, one-way ANOVA was conducted, and multiple comparisons were assessed using Duncan’s test. Significance was accepted at *p* < 0.05. Data are presented as mean ± standard deviation (mean ± SD), and relevant figures were plotted with GraphPad Prism 8.

## 3. Results

### 3.1. Growth Assessment

Data in Table 3 indicate that FBW, WGR, and SGR in the SCE10 group were comparable to the CT and SCC01 groups but were significantly lower than in the SCE05 group, whereas the SCE05 group itself showed no significant difference from the CT and SCC01 groups. In addition, the SCE10 group exhibited significantly higher feed FCR and FI compared to the other groups, while its PER was significantly lower. As for the biometric parameters, the VSI of the experimental groups was lower than the CT group (*p* > 0.05), and the HSI of the experimental groups was significantly lower than CT group (*p* < 0.05). In the composition of the whole body, the crude protein in the SCE groups significantly increased compared with the other groups (*p* < 0.05). Compared with the CT group, the crude ash content was significantly higher in the experimental groups (*p* < 0.05). As for the liver, the crude lipid level in the SCE groups was significantly lower than that of the other groups (*p* < 0.05).

### 3.2. Serum Biochemical Indices

The serum biochemical indices are shown in Table 4. Compared with the CT group, the levels of TG, TC, LDL-C and non-HDL-C in the three groups were significantly decreased, whereas the HDL-C/LDL-C ratio was significantly increased (*p* < 0.05). The levels of HDL-C remained stable (*p* > 0.05). As for the immunity parameters, TP was significantly higher (*p* < 0.05) in the SCE05 and SCE10 groups than in the CT and SCC01 groups. No significant differences were observed for ALB, LZM, AST, or ALT.

### 3.3. Enzymatic Activities

**Figure 1:**
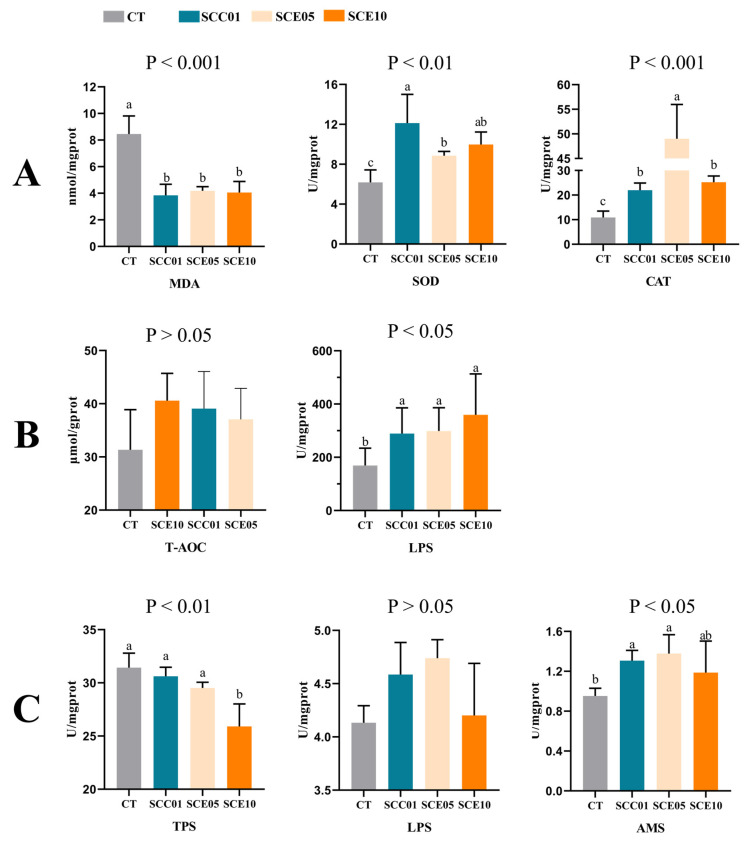
Hepatic and intestinal enzyme activity of common carp fed with experimental diets (*n* = 3). (**A**,**B**) Enzyme activity of liver, (**C**) enzyme activity of intestine. Columns with different superscripts are significantly different (*p* < 0.05). The lack of superscript letter indicates no significant differences among groups (*p* > 0.05). MDA (malondialdehyde); SOD (superoxide dismutase); CAT (catalase); T-AOC (total antioxidant capacity); LPS (lipase); TPS (trypsin); AMS (amylase).

After ingestion of the SCC and SCE diets by the fish, the impacts on responses of enzymatic activities are shown in Figure 1. The activities of CAT, SOD and LPS in the liver were significantly increased across all experimental groups (*p* < 0.05). The activities of MDA in the liver significantly decreased across all experimental groups (*p* < 0.05). The experimental groups exhibited elevated T-AOC in the liver, but this increase did not reach statistical significance (*p* > 0.05). Intestinal TPS activity demonstrated a significant decreasing trend in the SCE10 group (*p* < 0.05). In contrast to the CT group, both the SCE05 and SCC01 groups demonstrated increased intestinal LPS and AMS activities. Although the rise in LPS activities was not statistically significant (*p* > 0.05), the enhancement in AMS activities was significant (*p* < 0.05). Furthermore, the SCE10 group showed a slight, though not statistically significant, decrease in both intestinal LPS and AMS activities compared to the SCE05 group.

### 3.4. Liver Histomorphology

**Figure 2:**
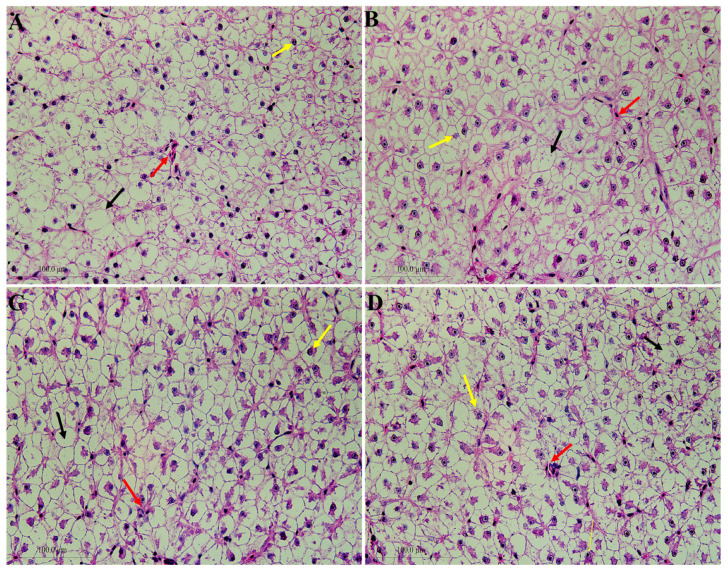
Hematoxylin and eosin (H&E) staining images for liver of common carp fed with experimental diets (*n* = 3). (**A**–**D**) CT, SCC01, SCE05, and SCE10 groups (400×), respectively. The red arrows represent congestion; the yellow arrows represent off-center hepatocyte nuclei; and the black arrows represent lipid vacuole.

**Figure 3:**
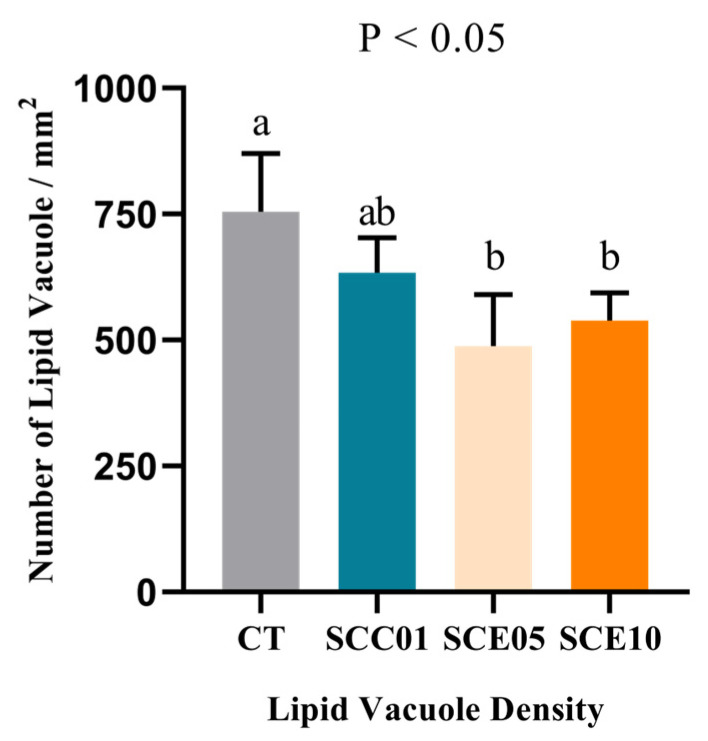
Lipid vacuole density in the liver of common carp fed with experimental diets (*n* = 3). Values with different superscripts in the same row are significantly different (*p* < 0.05).

Histopathological assessment of liver sections (Figure 2) revealed marked improvements in all experimental groups compared to the CT group, characterized by reduced diminished steatosis, fewer lipid droplets and clearer cellular boundaries. These morphological observations were substantiated by quantitative morphometry, which demonstrated a significantly lower density of lipid vacuoles across treatment groups (Figure 3). Notably, the SCE05 and SCE10 groups exhibited the most pronounced reduction in hepatic lipid accumulation relative to the CT group, confirming the lipid-lowering effect at the histological level.

### 3.5. Gene Expression Results

**Figure 4:**
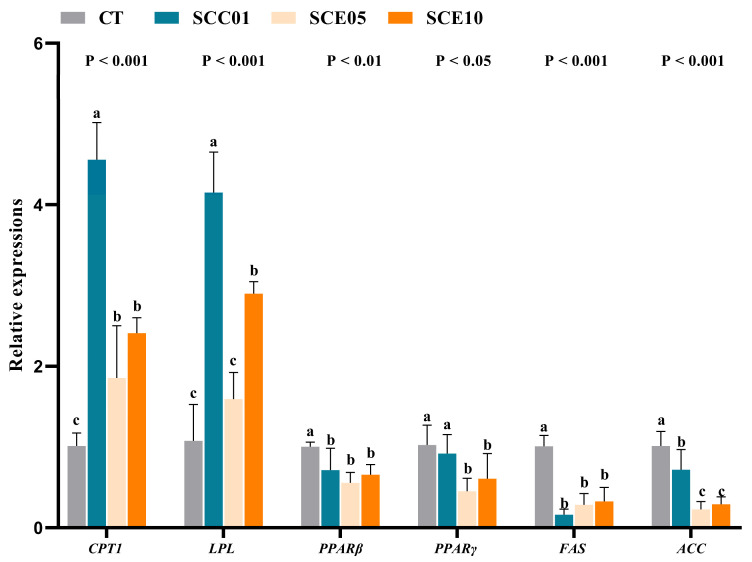
Liver relative expression levels of fat-metabolism-related genes in common carp fed with experimental diets (*n* = 3). Values with different superscripts in the same row are significantly different (*p* < 0.05).

The liver expression levels of lipid metabolism genes are shown in Figure 4. Compared with the CT group, the expression levels of *CPT1* were significantly up-regulated in the experimental groups (*p* < 0.05), the expression levels of *LPL* were significantly up-regulated in the SCC01 and SCE10 groups (*p* < 0.05), the expression levels of *PPARβ*, *FAS* and *ACC* were significantly down-regulated in the SCC01 and SCE10 groups (*p* < 0.05), and the expression levels of *PPARγ* were significantly down-regulated in the SCE groups (*p* < 0.05).

**Figure 5:**
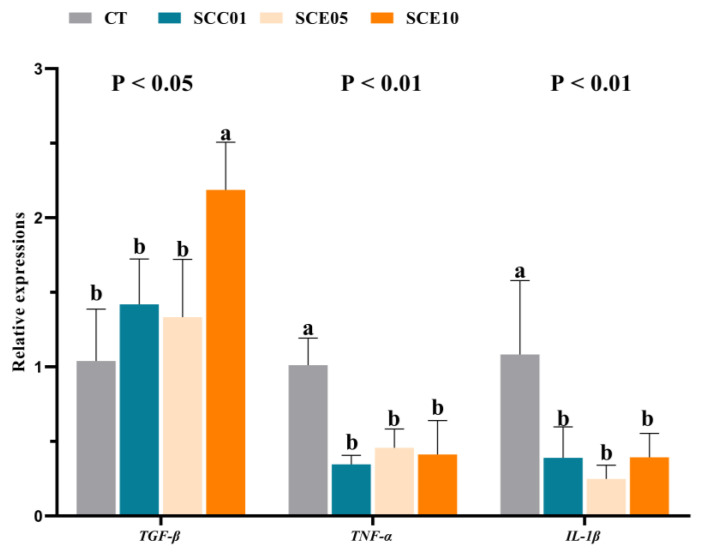
Liver relative expression levels of inflammation-related genes in common carp fed with experimental diets (*n* = 3). Values with different superscripts in the same row are significantly different (*p* < 0.05).

The liver expression levels of immunity genes are shown in Figure 5. Compared with the CT group, the expression levels of *TNF-α* and *IL-1β* were significantly down-regulated in the experimental groups (*p* < 0.05), and the expression levels of *TGF-β* were significantly up-regulated in the SCE10 groups (*p* < 0.05).

**Figure 6:**
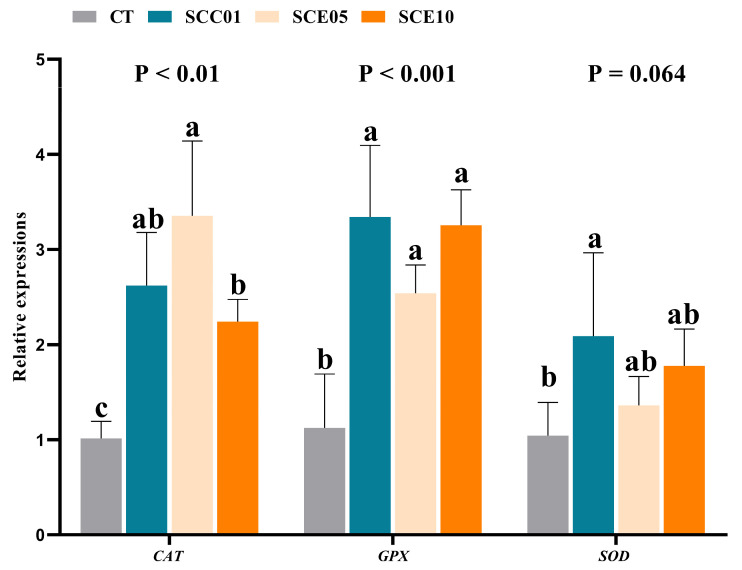
Liver relative expression levels of antioxidant genes in common carp fed with experimental diets (*n* = 3). Values with different superscripts in the same row are significantly different (*p* < 0.05).

The liver expression levels of antioxidation genes are shown in Figure 6. Compared with the CT group, the expression levels of *CAT* and *GPX* were significantly up-regulated in the experimental groups (*p* < 0.05), the expression levels of *SOD* were significantly up-regulated in the SCC01 group (*p* < 0.05).

## 4. Discussion

### 4.1. Growth Assessment

This study revealed a distinct dose-dependent effect of SCE supplementation on growth performance in common carp. While the inclusion of 0.5% SCE showed no adverse effects, 1% SCE supplementation significantly suppressed growth, as evidenced by the lowest values of FBW, WGR and SGR. This growth impairment was accompanied by increased FI and FCR, along with reduced PER and digestive enzyme activity (LPS, TPS and AMS), indicating disrupted feeding efficiency and nutrient utilization. Notably, similar phenomena have been observed with fermented ML and fermented SE [10,36], supporting the broader applicability of this dose–response pattern. The adverse effects observed at the 1% SCE supplementation level may be attributed to elevated dietary levels of anti-nutritional factors (ANFs) and non-starch polysaccharides (NSPs)—such as tannins, phytic acid, cellulose, and pectin—which are reported to be abundant in the source material SE [10,37,38]. Substantial evidence has demonstrated that these components not only adversely affect fish growth but also significantly reduce PER while increasing FCR [39,40,41,42]. In this study, the significant reduction in PER and elevation in FCR at the 1% SCE level are likely due to the marked inhibition of protease activity (e.g., TPS) compared to other groups, which impaired protein digestion and utilization, ultimately leading to the observed growth retardation in common carp. The lack of negative growth impacts at lower SCE concentrations (e.g., 0.5%) is likely attributable to the beneficial synergy of polysaccharides and minerals (e.g., calcium) present in SCE [3,43,44]. In contrast, excessive supplementation allows the detrimental effects of ANFs and NSPs to dominate. Compared to the dose-dependent effect observed with SCE, the inclusion of 0.1% SCC did not exert a significant influence on carp growth. This result may be attributed either to the insufficient dosage administered or to the possibility that chlorophyll derivatives such as SCC inherently lack a growth-promoting effect in this species.

Variations in fish proximate composition were driven by differential nutrient intake and directly impacted morphological parameters [45], which serve as reliable biomarkers for growth performance and dietary adaptation. In this study, the crude lipid content in the whole body, muscle and liver of the experimental groups showed significant reductions, paralleled by significant reductions in HSI. These findings demonstrate that SCE enhances lipid metabolism efficiency in common carp, thereby reducing hepatic lipid deposition. Previous studies have observed that 6% dietary ML significantly reduced the whole-body lipid content in largemouth bass, while 10% dietary fermented ML markedly decreased both whole-body lipid content and HSI in crucian carp (*Carassius auratus*)—findings consistent with the experimental results [36,46]. Furthermore, the results obtained from the SCC group demonstrate that SCC is capable of enhancing lipid metabolic efficiency in carp. Based on this finding, it can be inferred that within the multi-component SCE, chlorophyll likely functions synergistically with other coexisting phytochemicals to collectively enhance lipid metabolism, thereby producing an effect comparable to that of SCC. Nevertheless, this hypothesized synergistic mechanism necessitates further experimental validation.

### 4.2. Lipid Metabolism

Serum activities of AST and ALT are important liver function indicators [47]. In the present experiment, there were no significant differences among all the groups, suggesting that different treatments have no effects on liver function. Serum lipid parameters—including TG, TC, HDL-C, and LDL-C—serve as key indicators of lipid metabolic status [48]. LDL-C promotes cholesterol deposition in blood vessels, and HDL-C facilitates its clearance from circulation; thus, the HDL-C/LDL-C ratio reflects the overall efficiency of cholesterol transport [49,50]. In this study, supplementation with either SCE or SCC significantly reduced serum levels of TG, TC, and LDL-C while markedly increasing the HDL-C/LDL-C ratio, indicating enhanced lipid metabolism [51,52]. Histological examination in the experimental groups exhibited reduced hepatic lipid infiltration and deposition, reflecting improved hepatic lipid metabolic capacity. This morphological improvement aligns well with reductions in serum TG, TC and LDL-C levels. These results are consistent with previously reported lipid-lowering effects of ML powder in common carp [29] and largemouth bass [53]. Notably, similar improvements in lipid profiles have been observed with SCC [54] and chlorophyll [55] in other model organisms, which corroborates the effects seen in the SCC group. Overall, these findings indicate that both SCE and SCC exert comparable effects in improving blood lipid profiles in common carp.

The molecular mechanisms underlying these physiological and biochemical improvements were further elucidated through hepatic gene expression analysis. The reduction in hepatic lipid deposition observed histologically can be mechanistically explained by a coordinated shift in the expression of key genes regulating lipid metabolism. Specifically, SCE supplementation suppressed the expression of lipogenic genes (including *PPARβ*, *PPARγ*, *FAS* and *ACC*) while enhancing the expression of genes involved in lipid catabolism (including *CPT1* and *LPL*). The down-regulation of *PPARγ* is of particular significance, as it indicates inhibition of adipocyte differentiation and proliferation, thereby directly contributing to reduced lipid storage in hepatic tissue [29]. This gene expression pattern is consistent with findings from other studies involving ML supplementation [29,56].

Notably, a comparative analysis with the SCC group provided deeper insight into the role of individual components. SCC induced a significantly stronger up-regulation of lipolytic gene expression compared to the SCE groups, but it showed no marked suppressive effect on lipogenic genes associated with lipid storage. This differential gene expression profile indicates that SCC primarily enhances lipid catabolism, whereas SCE exerts a more comprehensive lipid-modulating effect that influences both lipid storage and breakdown pathways. Thus, although both compounds promote lipid metabolism, their underlying molecular mechanisms appear to be distinct.

### 4.3. Immunomodulatory Effects

Serum immune parameters (TP, ALB, and LZM) form essential primary defenses against pathogens. Compared to the control, SCE supplementation increased TP, indicating enhanced innate immunity [57,58]. Similar immunostimulatory effects have been documented with fermented SE in grass carp and common carp [10]. Given the distinct immune response patterns observed between the SCE and SCC groups, it is speculated that these two additives may function through different immunomodulatory mechanisms, with SCC likely exhibiting a comparatively limited effect in enhancing the overall immunity of common carp.

At the gene level, hepatic inflammatory responses are closely linked to immune function and are primarily regulated by pro-inflammatory cytokines (*IL-1β* and *TNF-α*) and anti-inflammatory cytokines (*TGF-β*) [59,60]. In this study, the expression levels of pro-inflammatory-related genes (*IL-1β* and *TNF-α*) were significantly down-regulated in the experimental groups. *TGF-β* gene expressions in SCE10 were significantly up-regulated when compared with the other three groups. These results demonstrate that SCE enhances the immune capacity of common carp by reducing the expression of pro-inflammatory genes in the liver, and higher levels of SCE further improve immune function by up-regulating anti-inflammatory genes. These gene-level findings collectively corroborate the earlier speculation that SCE and SCC modulate immunity through distinct pathways: SCE exhibits a coordinated, multi-targeted regulatory effect, while the immunomodulatory influence of SCC appears more confined.

### 4.4. Antioxidant Capacity

Reactive oxygen species (ROS) generated during fish development induce oxidative stress and impair antioxidant defense [60,61]. Key indicators of oxidative status include T-AOC, the activities of antioxidant enzymes such as SOD and CAT, and MDA level, a marker of lipid peroxidation and cellular damage [62,63,64]. In this study, SOD and CAT activity increased significantly and MDA decreased significantly in all treatment groups, suggesting a significant enhancement of systemic antioxidant capacity. It is noteworthy that the SCC01 group demonstrated a more marked elevation in SOD activity compared to the SCE groups, while a more pronounced increase in CAT activity was observed in the SCE05 group relative to the SCC treatment. At the molecular level, hepatic gene expression of *CAT* was elevated across all experimental groups, with a significant increase observed only in the SCE10 group. Similarly, while *SOD* expression was generally up-regulated, a significant elevation was detected only in the SCC01 group. These gene expression patterns are consistent with the corresponding changes in hepatic antioxidant enzyme activities. This aligns with previous studies reporting improved antioxidant status in crucian carp supplemented with fermented ML or its extracts [36,65]. Furthermore, natural chlorophyll and SCC have documented antioxidant properties, though related research has been limited in recent years [12]. The comparable overall antioxidant response observed in both the SCE and SCC groups—reflected in enhanced enzyme activities and elevated levels of corresponding antioxidant genes—indicates that their mechanisms likely involve overlapping pathways. However, the distinct patterns in specific enzyme activities and antioxidant gene expression suggest that their underlying antioxidant networks are not entirely identical.

## 5. Conclusions

This study demonstrates that both silkworm excrement concentrated extract (SCE) and sodium copper chlorophyllin (SCC) are effective functional additives for common carp, yet they exhibit distinct profiles. SCE supplementation showed a dose-dependent effect on growth, with 1% inclusion impairing performance, likely due to anti-nutritional factors, whereas 0.1% SCC did not significantly influence growth. Both additives significantly improved lipid metabolism but through different mechanisms: SCC primarily enhanced lipid catabolism, while SCE exerted a more comprehensive regulatory effect on both synthesis and breakdown pathways. Furthermore, SCE demonstrated superior, multi-targeted immunomodulatory capacity by modulating inflammatory cytokine expression, an effect not observed with SCC. Although both groups showed comparable overall antioxidant enhancement, distinct patterns in specific enzyme activities and gene expression indicated their underlying pathways are not identical. In conclusion, SCE offers broad-spectrum, synergistic benefits suitable for holistic health management, while SCC provides stable, specific bioactivity for targeted applications, highlighting the importance of additive selection based on desired functional outcomes in aquaculture.

## Figures and Tables

**Table 1:** Diet formulation and composition of experimental diets fed to common carp.

Ingredients (%)	CT	SCC01	SCE05	SCE10
Fish meal	12.7	12.7	12.7	12.7
Chicken meal	9.1	9.1	9.1	9.1
Krill meal	13.6	13.6	13.6	13.6
Shrimp shell powder	2.3	2.3	2.3	2.3
Peanut bran	13.6	13.6	13.6	13.6
Soybean meal	18.1	18.1	18.1	18.1
Fish oil	2.7	2.7	2.7	2.7
Spirulina	2.3	2.3	2.3	2.3
Vitamin mineral mixture ^1^	0.9	0.9	0.9	0.9
High-gluten flour	22.7	22.7	22.7	22.7
Carboxymethylcellulose sodium	2.0	1.9	1.5	1.0
SCC/SCE	0.0	0.1	0.5	1.0
Total	100.0	100.0	100.0	100.0
Proximate composition (%)				
Moisture (%)	8.6	8.6	8.5	8.5
Crude protein (%)	39.3	39.5	39.4	39.4
Crude lipid (%)	5.2	5.2	5.2	5.2
Ash (%)	12.5	12.8	12.6	12.8

Note: Data of composition are the means of three replicate measurements. ^1^ Vitamin premix provided the following per kg of diets: VA 50 mg, VB1 20 mg, VB2 30 mg, VB6 25 mg, VB12 0.08 mg, VD 25 mg, VE 80 mg, VK3 20 mg, folic acid 6.4 mg, niacin 150 mg, inositol 200 mg, calcium pantothenate 80 mg, biotin 0.32 mg, moisture ≤ 10%. Mineral premix provided the following per kg of diets: K 180 mg, Ca 1150 mg, Mg 50 mg, Zn 30 mg, Fe 50 mg, Mn 9.5 mg, Co 1.25 mg, Se 0.25 mg, moisture ≤ 10%.

**Table 2:** Primers used for real-time PCR gene expression assays.

Gene	Forward Primer	Reverse Primer	Source
*PPARβ*	GAGGCCATATTTGCGATGCTC	TCTCTCGTCACAAAGCCCTTC	[29]
*PPARγ*	TGCAAGGGATTCTTCCGCAG	AACGAATGGCGTTGTGTGAC	[30]
*CPT1*	CAGATGGAAAGTGTTGCTAATGAC	TGTGTAGAAGTTGCTGTTGACCA	[31]
*LPL*	CCCGATACCTTCCACATCCG	TTTTTGCGGCTTGACTGAGC	[29]
*ACC*	GTCACTGGCGTATGAGGATATT	TCCACCTGTATGGTTCTTTGG	[31]
*FAS*	GACAGGCCGCTATTGCTATT	TGCCGTAAGCTGAGGAAATC	[31]
*TNF-α*	ACTTCCAAGGCAGCCATCCATTT	TCAAGCCGCCTGAAGTGAAAGCC	[17]
*TGF-β*	CAGCACAAGAGCCACAGACAGAA	GCACTCCACAGATACGGACAGG	[17]
*IL-1β*	CAAACTGGAGCTGTCTTCGC	CTTCACCAGACGCTCTTCGAT	[32]
*CAT*	CTGACCCCGATTACTCCATCAG	GGGTTTCGGTTCAACACAAGG	[33]
*GPX*	TGTCCGCGAAACTATTGTCAG	GTTCATCTGGGTGTAATCCCT	[33]
*SOD*	TGAGCTGTCGGAAGCCATCAAG	TTGGTTCCCACATGCAGCAATCC	[34]
*β-actin*	TGAAGATCCTGACCGAGCGT	GGAAGAAGAGGCAGCGGTTC	[35]

Note: *PPARβ*, Peroxisome Proliferator-Activated Receptor Beta; *PPARγ*, Peroxisome Proliferator-Activated Receptor Gamma; *CPT1*, Carnitine Palmitoyltransferase1 I; *LPL*, Lipoprotein Lipase; *ACC*, Acetyl CoA Carboxylase; *FAS*, Fatty Acid Synthase; *TNF-α*, Tumor Necrosis Factor Alpha; *TGF-β*, Transforming Gowth Factor Beta1; *IL-1β*, Interleukin-1 Beta; *CAT*, catalase; *GPX*, Glutathione Peroxidase; *SOD*, superoxide dismutase.

**Table 3:** Growth performance, morphological parameters and proximate composition of common carps fed experimental diets.

Items	CT	SCC01	SCE05	SCE10	Anova *p* > F
IBW (g)	2.01 ± 0.08	2.02 ± 0.05	1.97 ± 0.11	1.98 ± 0.15	0.898
FBW (g)	28.03 ± 1.14 ^ab^	27.71 ± 1.23 ^ab^	29.14 ± 1.72 ^a^	25.92 ± 0.66 ^b^	0.073
WGR (%)	1294.39 ± 56.89 ^ab^	1271.59 ± 60.66 ^ab^	1379.12 ± 87.49 ^a^	1209.26 ± 33.51 ^b^	0.059
SGR (%/day)	3.14 ± 0.05 ^ab^	3.12 ± 0.05 ^ab^	3.21 ± 0.07 ^a^	3.07 ± 0.08 ^b^	0.118
FI (%/day)	2.78 ± 0.10 ^b^	2.84 ± 0.12 ^b^	2.83 ± 0.15 ^b^	3.13 ± 0.07 ^a^	0.024
FCR	1.35 ± 0.06 ^b^	1.38 ± 0.07 ^b^	1.36 ± 0.08 ^b^	1.53 ± 0.04 ^a^	0.027
PER (%)	187.88 ± 8.13 ^a^	184.03 ± 8.63 ^a^	186.35 ± 11.59 ^a^	165.31 ± 4.56 ^b^	0.040
SR (%)	95.55 ± 3.85	96.67 ± 3.34	98.89 ± 1.93	95.56 ± 5.09	0.672
CF (g/cm^3^)	2.93 ± 0.22	2.90 ± 0.33	2.97 ± 0.22	3.03 ± 0.17	0.333
VSI (%)	9.64 ± 0.75	8.94 ± 1.86	9.02 ± 1.09	8.68 ± 0.99	0.734
HSI (%)	2.42 ± 0.44 ^a^	1.92 ± 0.23 ^b^	1.90 ± 0.15 ^b^	1.83 ± 0.17 ^b^	0.018
Whole-body composition					
Moisture (%)	69.54 ± 0.75	70.83 ± 0.73	69.68 ± 1.51	70.53 ± 1.19	0.443
Crude protein (%)	15.55 ± 0.30 ^b^	15.68 ± 0.29 ^b^	16.49 ± 0.40 ^a^	16.43 ± 0.16 ^a^	0.008
Crude lipid (%)	12.47 ± 0.12 ^a^	10.82 ± 0.26 ^b^	10.46 ± 0.25 ^c^	9.91 ± 0.16 ^d^	0.003
Ash (%)	1.74 ± 0.06 ^c^	1.89 ± 0.04 ^b^	1.91 ± 0.04 ^b^	2.19 ± 0.04 ^a^	0.000
Liver composition					
Moisture (%)	67.56 ± 1.55	66.22 ± 2.66	65.74 ± 1.57	66.35 ± 2.000	0.721
Crude protein (%)	13.78 ± 0.14	14.14 ± 0.84	14.13 ± 0.40	13.79 ± 0.38	0.712
Crude lipid (%)	11.00 ± 0.11 ^a^	10.33 ± 0.04 ^b^	10.46 ± 0.03 ^b^	9.30 ± 0.05 ^c^	0.000
Ash (%)	1.66 ± 0.13	1.58 ± 0.10	1.69 ± 0.16	1.56 ± 0.13	0.561

Note: IBW (initial body weight), FBW (final body weight), WGR (weight gain rate), SGR (specific growth rate), FCR (feed conversion ratio), FI (food intake), PER (protein efficiency rate), SR (survival rate), CF (condition factor), VSI (viscerasomatic index), and HSI (hepatosomatic index). The proximate composition (crude protein, crude lipid, and ash) of the whole body and liver presented in the table was determined on a wet-weight basis. Values with different superscripts in the same row are significantly different (*p* < 0.05). The lack of superscript letters or rows with the same superscripts letter indicates no significant differences between groups (*p* > 0.05).

**Table 4:** Serum biochemical indices of common carps fed experimental diets.

Items	CT	SCC01	SCE05	SCE10	Anova *p* > F
Blood lipid parameters					
TG (mmol/L)	2.97 ± 0.34 ^a^	2.07 ± 0.38 ^b^	2.36 ± 0.05 ^b^	2.43 ± 0.21 ^b^	0.025
TC (mmol/L)	6.57 ± 0.57 ^a^	5.42 ± 0.52 ^b^	5.44 ± 0.13 ^b^	5.80 ± 0.20 ^b^	0.023
HDL-C (mmol/L)	2.73 ± 0.07	2.82 ± 0.17	2.81 ± 0.20	2.86 ± 0.06	0.711
LDL-C (mmol/L)	2.02 ± 0.35 ^a^	1.49 ± 0.13 ^b^	1.47 ± 0.09 ^b^	1.39 ± 0.24 ^b^	0.033
Non-HDL-C (mmol/L)	3.84 ± 0.62 ^a^	2.60 ± 0.43 ^b^	2.63 ± 0.07 ^b^	2.94 ± 0.14 ^b^	0.014
HDL-C/LDL-C	1.27 ± 0.09 ^b^	1.90 ± 0.09 ^a^	1.92 ± 0.20 ^a^	2.11 ± 0.4 ^a^	0.012
Immunity parameters					
TP (g/L)	28.74 ± 0.68 ^b^	28.34 ± 0.62 ^b^	30.18 ± 0.45 ^a^	31.88 ± 0.88 ^a^	0.008
ALB (g/L)	12.38 ± 0.22	13.05 ± 0.82	12.89 ± 0.66	13.24 ± 0.17	0.318
LZM (μg/mL)	0.56 ± 0.10	0.61 ± 0.08	0.63 ± 0.12	0.64 ± 0.15	0.842
Hepatic function					
AST (U/L)	400.72 ± 36.87	405.68 ± 35.44	419.54 ± 97.20	379.32 ± 35.00	0.858
ALT (U/L)	42.55 ± 9.29	41.48 ± 8.32	40.58 ± 14.10	39.65 ± 7.49	0.986

Note: TG (triglyceride), TC (total cholesterol), HDL-C (high-density lipoprotein cholesterol), LDL-C (low-density lipoprotein cholesterol), non-HDL-C (non-high-density lipoprotein cholesterol), TP (total protein), ALB (albumin), LZM (lysozyme), ALT (alanine aminotransferase), and AST (aspartate aminotransferase). Same row means with different superscripts differ significantly (*p* < 0.05). The lack of superscript letters or rows with the same superscripts letter indicates no significant differences between groups (*p* > 0.05).

## Data Availability

The original contributions presented in the study are included in the article. Further inquiries can be directed to the corresponding author.
